# Soft-tissue Sarcoma Survival in the US Military Health System: Comparison With the SEER Program

**DOI:** 10.5435/JAAOSGlobal-D-22-00122

**Published:** 2022-06-21

**Authors:** Ashley B. Anderson, Amie B. Park, Kangmin Zhu, Jie Lin, Craig D. Shriver, Benjamin K. Potter

**Affiliations:** From the Department of Surgery, Uniformed Services University of the Health Sciences and Walter Reed National Military Medical Center (Dr. Anderson, Dr. Park, Dr. Zhu, Dr. Lin, Dr. Shriver, and Dr. Potter); the Henry M. Jackson Foundation for the Advancement of Military Medicine (Dr. Park, Dr. Zhu, and Dr. Lin); the John P. Murtha Cancer Center Research Program, Uniformed Services University of the Health Sciences and Walter Reed National Military Medical Center (Dr. Zhu, Dr. Lin, and Dr. Shriver); and the Department of Preventive Medicine and Biostatistics (Dr. Zhu and Dr. Lin), Uniformed Services University of the Health Sciences.

## Abstract

**Introduction:**

The US Military Health System (MHS) provides universal health care to beneficiaries. Few studies have evaluated the potential influence of access to universal care on survival outcomes for sarcoma. This study compared the survival of adult patients with soft-tissue sarcoma in the MHS with the US general population.

**Methods:**

MHS data were obtained from the Department of Defense Automated Central Tumor Registry (ACTUR). US population data were obtained from the National Cancer Institute's Surveillance, Epidemiology, and End Results registry. Patients who were 25 years or older with a histologically confirmed musculoskeletal soft-tissue sarcoma were matched based on age, sex, and race. Kaplan-Meier survival curves and Cox proportional hazards models were used to compare 5-year survival in the two groups.

**Results:**

Adult patients in ACTUR had markedly lower 5-year mortality for soft-tissue sarcomas (hazard ratio=0.82; 95% confidence interval, 0.73 to 0.92) after adjustment for potential confounders. Lower 5-year mortality was found in most demographic subgroups for ACTUR patients compared with Surveillance, Epidemiology, and End Results patients.

**Conclusion:**

Five-year survival in the MHS compared with the US general population may suggest an important role of universal health care in improving the survival of patients with soft-tissue sarcoma.

Sarcomas make up a heterogeneous group of malignant neoplasms of mesenchymal origin and account for less than 1% of all new cancer diagnoses in adults annually.^1,2^ There are numerous histologic types, and all are rare to extremely rare. Sarcomas can be classified into two broad categories: (1) soft-tissue sarcomas and (2) primary sarcomas of the bone. There were an estimated 1.76 million new cancer diagnoses in 2019 in the United States, of which only 12,750 were soft-tissue sarcoma.^3^

The 5-year overall (all-cause) survival rate for soft-tissue sarcoma in the United States (US) is 65%.^3^ The time to primary care evaluation and early initiation of subsequent treatment are important predictors of outcomes.^4,5,6^ In universal healthcare systems, where out-of-pocket costs are low, patients may be more likely to seek primary care; however, wait time from referral to evaluation may be a barrier to access in some healthcare settings. A recent analysis of a large cancer registry in the United States found that low-income patients (those with no health insurance or no insurance or Medicaid) with soft-tissue sarcomas had decreased overall survival, regardless of the disease stage at presentation.^7^ Vasta et al^8^ found better overall survival for soft-tissue sarcomas and equivalent survival in bone sarcomas when comparing military with civilian pediatric patients. However, to the best of our knowledge, no studies have compared access to care or insurance status in relation to sarcoma survival for adult beneficiaries in the Military Health System (MHS).

The MHS is one of the largest health systems in the United States. It provides healthcare services to 9.4 million eligible active duty service members, National Guard members, reservists, and retirees both in nearly 700 military hospitals and clinics around the world through the TRICARE health plan.^9^ The MHS differs from the US general population in its universal care provided to its beneficiaries across the spectrum of diagnosis, treatment, and follow-up. Previous work found that universal care and cancer-specific care programs were associated with improved survival for patients with lung cancer^10^ and glioma.^11^ However, it is unknown whether the universal health care in the MHS affects clinical outcomes for soft-tissue sarcomas when compared with the US general population. Differences in sarcoma survival in these populations may suggest advantages of universal health systems in sarcoma care and outcomes. The purpose of this study was to compare the survival of patients with soft-tissue sarcomas in the MHS with that of the US general population. Patients with soft-tissue sarcoma in the MHS were hypothesized to have better survival than patients in the US general population.

## Methods

### Data Source

Deidentified data from the Department of Defense Automated Central Cancer Registry (ACTUR) and the data for public use from the National Cancer Institute's Surveillance, Epidemiology, and End Results (SEER) program were used for this study. The Institutional Review Board of Walter Reed National Military Medical Center approved the use of the database for this research project. Established in 1986, ACTUR is a data collection and clinical tracking system for active duty military personnel, retirees, activated national guards and reservists, and their dependents diagnosed and/or treated at military treatment facilities (or hospitals) in the MHS.^10,11^ It follows all patients for vital status following the Commission on Cancer's Facility Oncology Registry Data Standards, using a variety of sources including but not limited to contact with patient or patient's family, contact with managing physician(s), program inpatient or outpatient services, and verification by death certificates.^12^ ACTUR also uses vital status data from the National Death Index and date of death from the Defense Enrollment Eligibility Reporting System for military beneficiaries. Data collected in ACTUR include demographics, tumor characteristics, cancer treatment, follow-up, vital status, and other variables.

The data from the SEER cancer registries represented the US general population.^13^ The SEER Program was initiated by the National Cancer Institute in 1973, and it collects information such as demographics, cancer diagnosis, tumor characteristics, diagnostic procedures, vital status, duration of follow-up, and other variables.^13^ We used the SEER 18, which includes 18 population-based cancer registries covering approximately 28% of the US population (Atlanta, Connecticut, Detroit, Hawaii, Iowa, New Mexico, San Francisco—Oakland, Seattle—Puget Sound, Utah, Los Angeles, San Jose—Monterey, Rural Georgia, the Alaska Native, Greater California, Greater Georgia, Kentucky, Louisiana, and New Jersey).^13^ The SEER program population is similar to the general US population for socioeconomic factors such as poverty and education.^13^

### Study Subjects

We defined soft-tissue sarcoma according to the WHO classification of soft-tissue tumors^14^ and using the cancer site codes and morphology codes based on the International Classification of Disease for Oncology, third edition.^2,14^ Final classifications for soft-tissue sarcomas included rhabdomyosarcoma, fibrosarcomas/peripheral nerve sheath tumors/other fibrous neoplasms, other specific soft-tissue sarcomas, and other unspecified soft-tissue sarcomas (Table 1).

**Table 1:** Soft-tissue Sarcoma Final Classification

Classification	WHO Classification	ICD-O-3 Histology Code
Rhabdomyosarcomas	Skeletal muscle tumors	8901, 8910, 8912, and 8920
Fibrosarcomas, peripheral nerve sheath tumors, and other fibrous neoplasms	Fibroblastic/myofibroblastic tumor	8810, 8811, 8814, 8815, 8825, 8832, and 8840
	Nerve sheath tumors	8921, 9540, 9542, 9561, 9571, and 9580
Other specified soft-tissue sarcomas	Adipocytic tumors	8850, 8851, 8852, 8853, 8854, 8855, and 8858
	So-called fibrohistiocytic tumors	8830 and 9252
	Smooth muscle tumors	8890
	Pericytic (perivascular) tumors	8711
	Vascular tumors	9120 and 9133
	Chondro-osseous tumors	9180 and 9240
	Tumors of uncertain differentiation	8800, 8842, 8940, 8982, 8990, 9040, 9041, 9043, 8804, 9581, 9044, 9231, 9260, 9364, 8806, 8963, 8714, and 9137
Unspecified soft-tissue sarcomas	Undifferentiated/unclassified sarcomas	8801, 8802, 8803, 8804, and 8805

Histologically confirmed soft-tissue sarcomas in adult patients diagnosed between January 1, 1987, and December 31, 2013, were identified from both ACTUR and SEER databases. Adult patients were defined as age older than 25 years.^3,15^ To minimize imbalance in demographic characteristics across cohorts, for each ACTUR patient, we randomly selected four SEER cases matched on age (±5 years), sex (male and female), and race (White, Black, Asian, and Pacific Islander, and Other/Unknown).

### Study Variables

Overall survival (all-cause mortality) was evaluated over the 5-year follow-up. Age at diagnosis, tumor stage, tumor grade, histology group, diagnosis year, and region of diagnosis were found to be confounding factors.

### Statistical Analysis

For each type of sarcoma, we compared the distribution of demographic and tumor characteristics between ACTUR and SEER patients using χ^2^ tests. Kaplan-Meier curves and log-rank tests were used to compare 5-year survival between patients from ACTUR and SEER. A multivariable Cox proportional hazards model for matched data was used to estimate hazard ratios and 95% confidence intervals for ACTUR compared with SEER, adjusted for potential confounders that were identified in bivariate analyses as associated with both population (ACTUR versus SEER) and survival. The proportional hazards assumption was graphically evaluated for predictors using log-log plots. No departure from proportional hazards was apparent for the cohort after adjustment for confounders included as model covariates. Survival time was calculated based on the follow-up period from the date of diagnosis to the date of death. Five-year survival was estimated based on the follow-up period ending at 5 years after the date of diagnosis. Survivors at the 5-year period or the end of the study period and those patients lost to follow-up were censored as of December 31, 2014. All statistical analyses were completed using Statistical Analysis System Version 9.4.

## Results

### Demographic and Tumor Features of the Comparison Populations

A total of 1962 patients with soft-tissue sarcoma were identified from ACTUR and matched with 7848 patients from SEER. The differences between the two populations in geographic, year-at-diagnosis, and tumor-stage distributions are summarized in Table 2. Variables included a high number of missing patients; nonetheless, the available date revealed that ACTUR patients were more likely to be diagnosed at stage I (29.6% versus 13.5%; *P* < 0.0001) and a classification of an unknown stage was less likely (35.9% versus 65.1%; *P* < 0.0001) than SEER cases. In the ACTUR, there was a lower percentage of patients with a diagnosis of high-grade undifferentiated tumors than the SEER patients (24.6% vs 32.2%; *P* < 0.0001).

**Table 2:** Demographic and Tumor Characteristics of Adult Sarcoma Patients 25 Years and Older Diagnosed During 1987 to 2013 From the ACTUR and SEER Registries

	Soft-tissue Sarcoma		
	ACTUR	SEER	*P*-Value
	N (%)	N (%)	
Age groups			0.98
25-34	447 (22.8)	1760 (22.4)	
35-49	547 (27.9)	2216 (28.2)	
50-64	598 (30.5)	2392 (30.5)	
65+	370 (18.9)	1480 (18.9)	
Sex			1.00
Male	1107 (56.4)	4428 (56.4)	
Female	855 (43.6)	3420 (43.6)	
Race			1.00
White	1394 (71.1)	5576 (71.1)	
Black	354 (18.0)	1416 (18.0)	
Asian or Pacific Islander	120 (6.1)	480 (6; 1)	
Other/Unknown	94 (4.8)	376 (4.8)	
Year of diagnosis			<0.0001
1987-1989	181 (9.2)	319 (4.1)	
1990-1994	447 (22.8)	696 (8.9)	
1995-1999	394 (20.1)	916 (11.7)	
2000-2004	316 (16.1)	1951 (24.9)	
2005-2009	337 (17.2)	2085 (26.6)	
2010-2013	287 (14.6)	1881 (24.0)	
Region of diagnosis			<0.0001
Northeast	54 (2.8)	1158 (14.8)	
South	1052 (53.6)	1379 (17.6)	
Midwest	127 (6.5)	956 (12.2)	
West	616 (31.4)	4355 (55.5)	
Other/Unknown	113 (5.8)	0 (0)	
Histology			<0.0001
Histology 1^a^	24 (1.2)	122 (1.6)	
Histology 2^b^	443 (22.6)	1925 (24.5)	
Histology 3^c^	1423 (72.5)	5246 (66.9)	
Histology 4^d^	72 (3.7)	555 (7.1)	
Stage			<0.0001
I	580 (29.6)	1063 (13.5)	
II	241 (12.3)	442 (5.6)	
III	262 (13.4)	578 (7.4)	
IV	175 (8.9)	659 (8.4)	
Unknown	704 (35.9)	5106 (65.1)	
Grade (two-tier)			<0.0001
Low	523 (26.7)	1888 (24.1)	
High	483 (24.6)	2530 (32.2)	
Unknown	956 (48.7)	3430 (43.7)	

a Soft-tissue sarcoma—rhabdomyosarcomas

b Soft-tissue sarcoma—fibrosarcomas, peripheral nerve sheath tumors, and other fibrous neoplasms

c Soft-tissue sarcoma—other specified soft-tissue sarcomas

d Soft-tissue sarcomaunspecified soft-tissue sarcomas

### Univariable Survival Analysis

**Figure 1:**
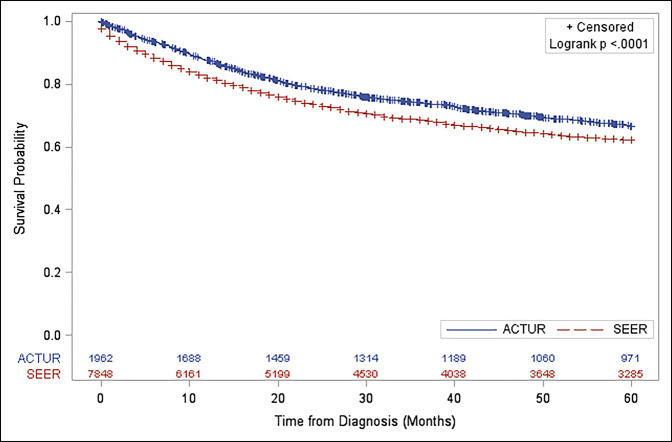
Graph showing Kaplan-Meier curves for 5-year survival of adult patients 25 years and older with soft-tissue sarcoma diagnosed during 1987 to 2013 from the ACTUR and SEER registries. ACTUR = Automated Central Tumor Registry, SEER = Surveillance, Epidemiology, and End Results.

The median overall survival times were 156 and 183 months for ACTUR and SEER patients, respectively. ACTUR patients compared with SEER patients showed significantly better 5-year Kaplan-Meier survival curves (log-rank *P* < 0.001) (Figure 1).

### Multivariable Survival Analysis

ACTUR patients had markedly better survival also based on the Cox proportional hazard model (adjusted hazard ratio = 0.82; 95% confidence interval, 0.73 to 0.92) than matched SEER patients after adjustment for age, year at diagnosis, and tumor characteristics (Table 3). This difference remained significant in the subgroups of 35 to 49 and 50 to 64-year-old patients, men and women, and White race (Table 3).

**Table 3:** Adjusted 5-year Survival Among Adult Sarcoma Patients 25 years and Older Diagnosed During 1987 to 2013 From the ACTUR and SEER Registries

	Soft-tissue Sarcoma			
	Alive	Dead	Adjusted HR^a^	95% CI
Overall				
SEER	5219	2629	1.00 (ref)	1.00 (ref)
ACTUR	1367	595	0.82	0.73-0.92
By age group				
25-34				
SEER	1386	374	1.00 (ref)	1.00 (ref)
ACTUR	371	76	0.79	0.55-1.14
35-49				
SEER	1643	573	1.00 (ref)	1.00 (ref)
ACTUR	427	120	0.76	0.59-0.98
50-64				
SEER	1481	911	1.00 (ref)	1.00 (ref)
ACTUR	388	210	0.81	0.66-0.98
65+				
SEER	709	771	1.00 (ref)	1.00 (ref)
ACTUR	181	189	0.90	0.72-1.12
By sex				
Male				
SEER	2965	1463	1.00 (ref)	1.00 (ref)
ACTUR	775	332	0.81	0.69-0.96
Female				
SEER	2254	1166	1.00 (ref)	1.00 (ref)
ACTUR	592	263	0.83	0.70-0.99
By race				
White				
SEER	3652	1924	1.00 (ref)	1.00 (ref)
ACTUR	962	432	0.83	0.72-0.95
Black				
SEER	938	478	1.00 (ref)	1.00 (ref)
ACTUR	253	101	0.77	0.58-1.02
Asian or Pacific Islander				
SEER	314	166	1.00 (ref)	1.00 (ref)
ACTUR	80	40	0.79	0.49-1.26
Other/Unknown				
SEER	315	61	1.00 (ref)	1.00 (ref)
ACTUR	72	22	1.05	0.43-2.57
By grade				
Low				
SEER	1600	288	1.00 (ref)	1.00 (ref)
ACTUR	455	68	1.07	0.62-1.83
High				
SEER	1189	1341	1.00 (ref)	1.00 (ref)
ACTUR	242	241	0.97	0.75-1.24
Unknown				
SEER	2430	1000	1.00 (ref)	1.00 (ref)
ACTUR	670	286	0.69	0.54-0.87
By stage				
Stage I				
SEER	898	165	1.00 (ref)	1.00 (ref)
ACTUR	506	74	0.64	0.30-1.39
Stage II				
SEER	339	103	1.00 (ref)	1.00 (ref)
ACTUR	180	61	0.72	0.11-4.76
Stage III				
SEER	379	199	1.00 (ref)	1.00 (ref)
ACTUR	140	122	1.27	0.48-3.33
Stage IV				
SEER	141	518	1.00 (ref)	1.00 (ref)
ACTUR	28	147	0.51	0.23-1.15
Stage unknown				
SEER	3462	1644	1.00 (ref)	1.00 (ref)
ACTUR	513	191	0.75	0.61-0.92
By histology				
Histology 1^b^				
SEER	46	76	1.00 (ref)	1.00 (ref)
ACTUR	5	19	—	—
Histology 2^c^				
SEER	1705	220	1.00 (ref)	1.00 (ref)
ACTUR	412	31	0.42	0.18-1.01
Histology 3^d^				
SEER	3182	2064	1.00 (ref)	1.00 (ref)
ACTUR	920	503	0.84	0.73-0.97
Histology 4^e^				
SEER	286	269	1.00 (ref)	1.00 (ref)
ACTUR	30	42	0.70	0.15-3.22

a The HR for the overall model was adjusted for age at diagnosis, tumor stage, tumor grade, histology, and year of diagnosis group. The HRs for stratified models were not adjusted for the stratifying variable (i.e. adjustment for the tumor stage was removed from the model when stratifying by stage), except age (as a continuous variable) was maintained in stratified models by the age group.

b Soft-tissue sarcoma—rhabdomyosarcomas

c Soft-tissue sarcoma—fibrosarcomas, peripheral nerve sheath tumors, and other fibrous neoplasms

d Soft-tissue sarcoma—other specified soft-tissue sarcomas

e Soft-tissue sarcoma—unspecified soft-tissue sarcomas

## Discussion

The US MHS population may differ from the US general population in both its exposure to cancer risk factors and access to medical care. Our study found that Department of Defense beneficiaries with soft-tissue sarcomas had 18% lower 5-year mortality compared with patients in the general population who had similar demographic characteristics. This finding contributes to the growing body of evidence suggesting that the improved survival may not be related to specific cancer diagnosis. Universal access to care in the MHS could be a contributing factor to the better survival observed.

To the best of our knowledge, no previous studies have evaluated clinical outcomes of soft-tissue sarcoma in the MHS compared with the general population. However, survival outcomes of other cancers have been compared in the two populations. Lin et al^10^ reported improved survival in patients with non–small-cell lung cancer and glioma in the MHS to have better overall survival than in the general population.^11^ In one of few studies in another universal healthcare system, the Veterans Health Administration (VHA) health system, Landrum et al found that survival rates for VHA patients with colon cancer were equivalent to or better than the survival rates of similar patients treated under fee-for-service Medicare. The improved survival rates in the VHA were explained in part by a higher frequency of cancer diagnoses at earlier stages.^16^ In the general population, worse survival has been documented in uninsured and Medicaid patients with cancer.^17^ Patients with non-Medicaid insurance had longer survival compared with patients with Medicaid insurance for primary bone sarcoma and extremity soft-tissue sarcoma as found by Smartt et al,^18^ using SEER data. The authors concluded that Medicaid patients have worse survival, possibly because of advanced disease at the time of presentation,^5,18,19^ and it is well known that both grade and stage at presentation affect survival.^6,20,21^ These results suggest that differences in insurance and access to cancer care may have an important effect on survival.^22^

There were several limitations to our study, including those which often apply to all cancer registry studies. First, we used death from all causes rather than sarcoma-related death as the study outcome because of incomplete information on the cause of death in the data. Because of lack of relevant information on comorbidities in the cancer registry data, we could not assess and do not exclude the potential effects of comorbid conditions on the overall survival, the primary study outcome. Second, there was a high proportion of missing grade and stage data in both study cohorts. The percentage of missing in grade data and other variables used to construct the four-tier American Joint Committee on Cancer (AJCC) stage variable was high in both the ACTUR and SEER data for our study samples, consistent with previous studies using the SEER database.^1,15,20,23,24^ We do not exclude potential confounding effects of unknown tumor stage and grade. Third, we acknowledge potential differences between the two cancer registries in the completeness of follow-up data. For example, some small military treatment centers might not have dedicated cancer registrars assigned to track patients with cancer, potentially limiting the completeness of outcome data for these catchment areas. Fourth, the military population is more mobile because of frequent changes in duty stations. Therefore, it is impractical to adjust for the potential effects of a region/area. In addition, follow-up times vary by diagnosis, but restricting the follow-up period limits the power of the analysis. Fifth, owing to the features of the cancer registry data, information on many factors such as comorbidities, body mass index, and tobacco use that may differ between the two populations and affect sarcoma survival was not available. Thus, we do not exclude the effects of residual confounding by unmeasured factors. Sixth, although we matched the two populations on race, we did not match them on ethnicity because of the less complete information on ethnicity for ACTUR. Thus, the potential effects of ethnicity on the results could not be excluded.

There have been no soft-tissue sarcoma studies that compare survival due to variations in treatment received, healthcare delivery, quality of care, or other factors between patients in a universal healthcare system and those in the general population. We speculate that the observed survival advantage among patients with soft-tissue sarcoma may have resulted from earlier diagnosis and timely treatment because of less barriers to medical care. However, differences between the military and civilian healthcare systems are likely multifactorial and related health characteristics that are not directly related to treatment. Additional work to describe care seeking and health characteristics in MHS patients is warranted to characterize the relative importance of access to universal care.

## Conclusion

We found that patients with soft-tissue sarcoma in the MHS have better survival than those in the US general population. These results suggest universal health care in the MHS may translate into improved survival for soft-tissue sarcoma.
